# Advances in the Study of the Potential Hepatotoxic Components and Mechanism of *Polygonum multiflorum*

**DOI:** 10.1155/2020/6489648

**Published:** 2020-09-30

**Authors:** He-Shui Yu, Lin-Lin Wang, Ying He, Li-Feng Han, Hui Ding, Xin-Bo Song, Xiu-Mei Gao, Nai-Ru Yun, Zheng Li

**Affiliations:** ^1^College of Pharmaceutical Engineering of Traditional Chinese Medicine, Tianjin University of Traditional Chinese Medicine, Tianjin 301617, China; ^2^Tianjin State Key Laboratory of Modern Chinese Medicine, Tianjin University of Traditional Chinese Medicine, Tianjin 301617, China; ^3^Second Affiliated Hospital of Tianjin University of Traditional Chinese Medicine, Tianjin 300250, China; ^4^Tianjin Modern Innovative TCM Technology Co., Ltd., Tianjin 300000, China

## Abstract

The roots of *Polygonum multiflorum* (PM) (He Shou Wu in Chinese) are one of the most commonly used tonic traditional Chinese medicines (TCMs) in China. PM is traditionally valued for its antiaging, liver- and kidney-tonifying, and hair-blackening effects. However, an increasing number of hepatotoxicity cases induced by PM attract the attention of scholars worldwide. Thus far, the potential liver injury compounds and the mechanism are still uncertain. The aim of this review is to provide comprehensive information on the potential hepatotoxic components and mechanism of PM based on the scientific literature. Moreover, perspectives for future investigations of hepatotoxic components are discussed. This study will build a new foundation for further study on the hepatotoxic components and mechanism of PM.

## 1. Introduction

The occurrence of adverse reactions caused by TCMs and their agents has increased, as they are widely used as disease treatments, in healthcare, etc. The liver, as an important drug-metabolizing organ, is particularly vulnerable to damage. PM, as a traditional tonic medicine, has antiaging, liver- and kidney-tonifying, hair-blackening, etc. effects [1–3].

However, PM-induced hepatotoxicity cases have been frequently reported in China and other countries. Hepatotoxicity cases caused by PM were reported by the Medicines and Healthcare Products Regulatory Agency (MHRA) [4–6]. Then, the China National Medical Products Administration (NMPA) also issued a notice on strengthening the supervision of health foods containing PM. Additionally, “Focus on Risk of Liver Damage Caused by Oral PM and Its Preparation” was published in “Adverse Drug Reaction Information Bulletin” by National Center for ADR Monitoring, China. As PM and its preparations are widely used in the treatment and prevention of diseases, the hepatotoxic effects of PM-based products limit its long-term usage, and the safety of PM requires attention.

The clinical hepatotoxic feature of PM was summarized according to the literature. The ingestion of PM formulations or preparations leads to liver injury, including PM soaked in wine after steaming, PM dried and ground into powder after steaming, oral liquid containing PM, capsules containing PM, Shou Wu tea, etc. PM and its preparations have been reported to lead to liver injury after injection for several days to several months and at a large range of doses. The ingestion of PM leads to liver injury in many populations, including the general population and people with hair loss, white hair, vitiligo, itchy skin, high blood pressure, coronary heart disease, cerebrovascular stenosis, high cholesterol, dermatitis, eczema, acne, and cerebral infarction. The clinical features of liver injury that result from the ingestion of PM or its preparations include nausea, vomiting, diarrhea, abdominal pain, nervous, restlessness, difficulty breathing, tic, upper gastrointestinal bleeding, hybrid hepatitis, cholestasis hepatitis, etc. The ingestion of PM leads to liver injury in people aged 5～78 years, and the occurrences in men and women are almost equal. In conclusion, the clinical features of liver injury that result from the ingestion of PM are not definitively related to processing, duration, dosage, age, gender, complications, etc. [4–10] (Table 1).

However, although PM extracts are generally considered to be relatively safe, PM extracts may still show hepatotoxicity. Most ancient books recorded that PM is not poisonous, but a small number of ancient books, such as “*Ben Cao Hui Yan*,” recorded that raw PM is qi cold and cold having property of contraction, and raw PM is poisonous. PPM is qi warm and not poisonous. These prove that PM contains compounds that could induce adverse reactions. Unfortunately, although extensive experiments have been performed both in vivo and in vitro in recent years, the potential toxic components and possible mechanisms that cause liver injury remain unclear.

The aim of this review is to provide comprehensive information on the potential hepatotoxic components and mechanism of PM based on the scientific literature. Moreover, perspectives for future investigations of hepatotoxic components are discussed. This study will build a new foundation for further study on the hepatotoxic components and mechanism of PM.

## 2. Study of Potential Hepatotoxic Compounds in PM

Modern research on PM begins with the study of its composition. Stilbenes, anthraquinones, procyanidins, flavonoids, phospholipids, catechins, and other compounds were isolated and identified from PM [15–17]. All these compounds play indispensable roles in the pharmacological effects of PM. However, scholars have begun to study potential hepatotoxic compounds, as frequent reports of liver injury involve PM. Anthraquinones (AQs), stilbenes, and catechins or their derivatives are the main controversial potentially toxic ingredients.

### 2.1. AQs or Their Derivatives and Liver Injury

AQs are one of the major potential hepatotoxic compounds in PM. The effects of raw PM and processed PM (PPM) with 70% ethanol extract on the liver were studied. These results showed that the 95% ethanol elution of the ethanol extract could inhibit the growth of L02 cells [18–20]. The 95% ethanol elution of raw PM and PPM ethanol extract are considered to be potential hepatotoxic parts of PM. In addition, researchers found that the order of toxicity was raw PM ethanol extract > raw PM water extract > PPM ethanol extract > PPM water extract [21–25]. It is worth noting that the content of AQs, such as emodin, and its derivatives were higher or main compounds in PM ethanol extract and 95% ethanol elution of PM ethanol extract [15], which reveals that AQs may be toxic ingredients. Therefore, the effect of main anthraquinone compounds, such as emodin, rhein, physcion, chrysophanol, etc., on the liver was studied in vivo and in vitro [26–38].

Studies have shown that emodin and rhein can deplete GSH, promote the production of intracellular ROS and the depolarization of the mitochondrial membrane, and upregulate the levels of cleaved Caspase-8, Caspase-9, Caspase-3, etc., inducing the apoptosis in L02 and HepG2 cells [39–43]. AQs and anthranone from PM, such as emodin and chrysophanol, significantly inhibited the activity of the bile salt export pump (Bsep), multidrug resistance-associated protein 2 (Mrp2), and basolateral efflux transporters; downregulated the activity of Na+/taurocholate cotransporting polypeptide (Ntcp) [44]; altered bile acid (BA) disposition; and resulted in liver injury. While anthraquinone and dianthrone exhibited the strongest inhibitory effect on UGT1A1 activity [34, 45, 46], UGT1A1 is an important UGT isoform involved in the metabolic clearance of bilirubin, which is a toxic waste product of heme degradation [47]. The inhibition of UGT1A1 may lead to bilirubin accumulation, which could induce jaundice, liver injury, etc. [48–50]. In addition, research has shown that the oral administration of emodin to rats can directly induce liver damage, and emodin can induce intracellular oxidative stress and ER stress through the AhR-CYP1A1 pathway and then induce the apoptosis pathways in hepatocytes [32, 33, 51].

The above results suggest that emodin, rhein, physcion, chrysophanol, emodin-8-O-glc, emodin-O-(acetyl)-hex, emodin-O-hex-sulfate, emodin-O-glc, emodin-O-(malonyl)-hex, and other anthraquinone components or their derivatives might be potential hepatotoxic compounds in PM.

However, some studies have noted that the speculation that AQs are the hepatotoxic components in PM lacks scientific evidence. Emodin, as well as other components, has a low bioavailability and might lead to liver damage only at high concentrations. According to the literature, to have liver damage, healthy adults need at least 339 g of PM in a single oral dose. However, the clinical dose of PM is 3～6 g of raw Shou Wu and 6～12 g of PPM [52, 53]. Thus, the hepatotoxic dose of AQs in PM is far from the actual dose, and the speculation that AQs are the hepatotoxic component of PM needs to be further explored.

### 2.2. Tannin and Liver Injury

Tannins can cause liver damage in grazing animals and were the main cause of cryptogenic liver damage in the past. The content of tannins in PM is high, up to 15%, and its content decreases as processing time increases [54]. Scholars have studied the role of tannins in liver damage caused by PM and found that tannins could significantly increase serum ALT, AST, AKP, TP, AIB, and TBA enzyme activity and induce significant damage in hepatocytes [55]. The administration of long-term high-doses and short-term medium doses is harmful to the liver in mice, and small doses induce no obvious liver damage. However, liver damage can be restored after stopping drug administration [56]. Research has also shown that gallic acid impaired the folding and processing of functional proteins, causing endoplasmic reticulum stress and generating liver cell apoptosis by repressing the biological control of the transcription and expression of peptidyl-prolyl *cis-trans*-isomerase A (PPIA) in a dose-dependent manner [57].

In addition, tannins induce the CYP2E1 enzyme [58], and previous studies concluded that the induction of CYP2E1 by tannin may affect the conversion and metabolism of AQs in vivo, which may lead to liver damage.

### 2.3. TSG (2,3,4′,5-Tetrahydroxystilbene-2-O-*β*-D-glucoside) and Liver Injury

Stilbenes, especially TSG, are considered to be the main liver-protective components in PM. However, research has found that TSG might be associated with the hepatotoxicity of PM.

Generally, the adverse effect of PM will diminish or vanish, and it has tonifying effect after processing; in other words, the hepatotoxicity of raw PM is higher than that of PPM [21–25]. Coincidently, studies have found that the content of *trans*-TSG in PPM was reduced by 55.8% and the content of emodin in PPM was increased by 34% compared with that in PM; that is, the content of *trans*-TSG was reduced and the content of emodin was increased during the processing. Therefore, some reports speculated that the toxicity of PM might not be correlated with the content of emodin or its derivatives but depended on the contents of *trans*-TSG, conventionally thought to be a liver-protective compound, or the relative content of *trans*-TSG and emodin [59, 60]. Reports have also shown that *trans*-TSG might inhibit the activation of the nuclear factor kB (NF-kB) signaling pathway, which is necessary for the HGF-mediated proliferation of WBF-344 cells. *Trans*-TSG may have some influence on the proliferation of liver cells [61–64].

*Trans*-TSG was unstable in irradiation and alkaline conditions and could convert to *cis*-TSG. Unfortunately, the latest research shows that *cis*-TSG has a higher cytotoxicity than *trans*-TSG in normal L02 cells, and *cis*-TSG could induce hepatotoxicity in LPS-treated rats. In addition, the contents of *cis*-TSG were higher in PM preparations or serum from patients with liver intoxication associated with PM than those in control samples, which may be because *trans*-TSG converted to *cis*-TSG in the course of preparation of PM and thus led to liver toxicity. All these results suggested that *cis*-TSG is closely associated with the hepatotoxicity of PM [65–67].

## 3. Hepatotoxic Mechanism of PM

In summary, the existing literature shows that the mechanism of PM-induced liver damage is mainly categorized into four viewpoints: (1) active substances in PM cause cholestasis, which leads to liver injury caused by lipid peroxidation or directly causes liver cell damage; (2) active substances in PM affect the CYP450 enzyme system, affecting drug transport or metabolism and leading to liver damage; (3) active substances in PM cause mitochondrial dysfunction, which leads to liver injury through the oxidative stress response; and (4) active substances in PM cause drug-induced liver injury (DILI).

### 3.1. Cholestasis and Liver Injury

 Cholestasis and bile duct injury are the main clinical manifestations of cholestasis caused by chemical drugs. Serum biochemical parameters, including alkaline phosphatase (ALP), total bilirubin (TBIL), and bile acid (TBA), are preferred in clinical practice. Studies have shown that the ALP, TBIL, and TBA were significantly different in the serum of rats after administration with PM compared with the serum of control rats [11, 22, 45], of which TBA increased in serum and increased in liver. Intrahepatic deposition of bile acids is considered to be the primary cause of cholestatic liver injury [68]. While cholic acid (CA), glycodeoxycholic acid (GDCA), chenodeoxycholic acid (CDCA), deoxycholic acid (DCA), and ursodeoxycholic acid were all significantly decreased, TCA was significantly increased, and GCA and GCDCA showed no significant changes [69]. These results indicated that the distribution of bile was also affected. The ethanol extract of PPM could affect the synthesis of bile acids and alter the composition of intestinal bile acids by activating Fxr-Fgf15 signaling in the intestine, and it can inhibit the expression of CYP7A1 in the liver [70]. In addition, AQs and dianthrones from PM, such as emodin and chrysophanol, significantly affected the activity of Bsep, Mrp2, and CYP8B1 and exhibited the strongest inhibitory effect on UGT1A1 activity, leading to bilirubin accumulation and resulting in cholestasis [34, 44–50, 71–73]. Meanwhile, hydrodeoxycholic acid (HDCA) in serum and tauro-*β*-muricholic acid (T*β*MCA) in urine were identified in rats with PM-induced liver injury [74–76]. Clinical analysis of drug-induced liver injury caused by PM or its preparations also indicated that there are many symptoms related to jaundice in patients with liver injury, including an increase in related indicators such as TBIL [7–10]. Therefore, liver injury caused by PM might be associated with cholestasis.

In the process of the generation, formation, transportation, and discharge of bile acid, failure at any step could cause cholestasis. When bile acid accumulation in liver cells or antioxidants decreases, the body will produces an oxidative stress reaction. This phenomenon could reduce the activity of superoxide dismutase (SOD), catalase (CAT), and glutathione peroxidase (GPx). First, ROS production increases; then, intestinal barrier dysfunction develops due to the lack of bile salts in the intestine. Next, excessive intestinal endotoxin (LPS) enters the systemic circulation, inducing the production of the inflammatory cytokines IL-1, IL-7, TNF-*α*, etc. and generating additional ROS, which further aggravate oxidative damage in the body [76–78].

Excessive ROS in the body cause peroxidative damage in liver cells by directly damaging biomacromolecules such as proteins and DNA [79, 80]. ROS act as direct functional signaling molecules, activate intracellular stress-sensitive signaling pathways, and mediate hepatocyte apoptosis. ROS induce hepatocyte apoptosis by interfering with mitochondrial function and indirectly by activating apoptotic signaling pathways. In addition, ROS can mediate inflammatory reactions and then cause liver damage [81, 82], which explains the mechanisms of hepatitis symptoms in patients with PM-induced liver injury [83, 84].

### 3.2. CYP450s and Liver Injury

The CYP450 enzymes, which are highly concentrated and exhibit activities in the human liver, are responsible for the oxidation or reduction of medicines [85, 86]. To date, more than 57 isoforms have been discovered in the CYP450 family; however, the CYP1A1, CYP1A2, CYP2B6, CYP2C8, CYP2C9, CYP2C19, CYP2D6, CYP2E1, CYP3A4, and CYP3A5 isoforms can metabolize nearly 90% of the drugs in the market [85]. It is well known that the activities of these CYP isoforms may be inhibited or induced by several compounds, and the modulation of their drug-metabolizing activities may have effects on pharmacodynamics and toxicology, such as DDI [86–88]. CYP450 and its isoenzymes are widely used in research fields such as drug metabolism, clinical rational drug use, toxicology, tumor biochemistry, and new drug research and development. In recent years, marked progress has been made in drug metabolism enzyme research.

Enzyme activity can be induced or inhibited by certain drugs to promote drug metabolism or to inhibit drug metabolism. If the metabolism of the drug inhibits the activity of a drug-metabolizing liver enzyme, the drug or its metabolites will accumulate in the liver and cause adverse reactions. The composition of traditional Chinese medicines is complex, but most TCM components are metabolized by the CYP450 enzyme and may further affect the metabolism or interaction of other drugs, such as the “eighteen incompatible medicaments,” in which the combined application of *Salvia*, *Sophora*, *Ginseng*, and gourd inhibits the activity of CYP3A and CYP2E1 [89]. The combined administration of aconite, melon, *Bletilla*, *Pinellia*, *Fritillaria*, and Radix Ampelopsis inhibited the activity of CYP3A and CYP1A2 [90]. The activity of these metabolic enzymes is altered to impair the metabolism of the drug in the body, producing toxic effects.

#### 3.2.1. CYP450 Inhibition

Research found that raw PM and PPM could significantly inhibit the expression of CYP2E1 mRNA [91]. The alcoholic extract of PM inhibited the activity of CYP2C19 and CYP2C9, and the aqueous extract of PM inhibited the activity of CYP2C19, CYP2C9, CYP2B6, CYP2D6, and CYP1A2 [92, 93]. TSG has inhibitory effects on mouse liver CYP1A2, CYP2E1, and CYP3A11 protein expression through the suppression of AhR, PXR, and PPAR*α* activation [94]. CYP1A2, 2C19, and 2E1 have been reported as the main CYP450s which participated in phase I metabolism of AQs such as emodin [92, 95]. Therefore, it is speculated that the mechanism by which PM induces liver injury may be that some components in PM inhibit the activity of CYP2E1, which causes the accumulation of AQs or other toxic substances metabolized by CYP2E1, causing liver damage.

#### 3.2.2. CYP450 Allelic Defect

The proportion of people with genetic defects in CYP450 enzymes is large [96]. However, due to the poor specificity of each CYP450 enzyme subtype substrate, each gene defect affects the metabolism of multiple drugs. Moreover, the incidence of different mutations that slow metabolism is different in different populations.

According to reports, whether CYP1A2 allele polymorphisms are associated with the acute liver injury induced by PM was tested by researchers using 43 cases of PM-induced liver injury. The results show that the frequency of the CYP1A21C allele was 46.5% in PM-induced DILI patients, which was significantly different from the frequency of 27.9% observed in healthy subjects. The frequency of the CYP1A21F allele was 63.9% in PM-induced DILI patients, compared to 57.0% in healthy controls; this difference was not significant. The allelic frequencies of CYP1A22, CYP1A27, CYP1A29, and CYP1A211 were not detected [97]. These results indicate that the CYP1A2 allelic mutation is most likely related to the metabolism of compounds in PM, followed by acute liver injury. Coincidentally, tannins, contained in rhubarb, are inducers of CYP2E1, which accelerate the conversion of CCl_4_ to CCl_3_, aggravating the liver injury caused by CCl_4_ [98]. Additionally, the activity of CYP450 may be related to the liver damage induced by PM.

### 3.3. Mitochondrial Damage and Liver Injury

Mitochondria are important organelles that generate energy, oxidize materials in cells, and play a fundamental role in energy metabolism, free radical production, aging, and apoptotic regulation. Possible factors leading to mitochondrial dysfunction include respiratory chain defects, metabolic enzyme inactivation, structural changes, and mutations. All of these factors affect the normal function of cells, resulting in the occurrence of diseases. Mitochondria are important targets of drug toxicity, and mitochondria are also the organelles most vulnerable to drug toxicity. The liver is also a major target of drug damage because it is an important organ in drug metabolism. Drugs induce liver injury primarily by changing the activity of enzymes, modulating the structure of mitochondria and/or decreasing the synthesis of mtDNA, further undermining *β*-oxidation and other oxidative processes in liver cells [99–101].

Research shows that rhein could be changed into a reactive metabolite by CYP2C19, and the structure of reactive metabolite was inferred to be an epoxide compound. The reactive metabolite covalently binds to intracellular mitochondria, leading to ROS overproduction and respiratory chain dysfunction. In addition, the reactive metabolite overproduction depletes GSH, which could result in hepatotoxicity [102–104]. Research also found that emodin could conjugate with GSH, forming emodin-GSH and depleting GSH in PM-induced liver injury rats [40–42]. An increasing number of studies have shown that mitochondrial damage may be a factor and pathway that predisposes individuals to PM-induced liver injury.

### 3.4. Drug-Induced Liver Injury

#### 3.4.1. Misuse of Counterfeit Goods

He Shou Wu, the roots of *Polygonum multiflorum*, was originally published in “Kai Bao Materia Medica” in the Northern Song Dynasty. This medicine has two kinds, red and white; the red type is commonly used in clinic. Common species used to as counterfeits of this medicine are *Musa basjoo* S. & Z., *Pteroxygonum giraldii Dammer & Diels*, *Polygonum ciliinerve* (Nakai) Ohwi, *Stephania cepharantha* Hayata, *Cynanchum auriculatum* Royle ex Wight, etc. In addition, PM has many origins, including Henan, Jiangxi, Guangdong, and Guangxi. The chemical content and efficacy of PM samples from different origins are also different [105–107]. Additionally, there is a large difference in quality. At present, due to insufficient market supervision, the confusion of origin and variety is also an important consideration in the safety of PM.

#### 3.4.2. Improper Processing

Records on the processing of PM include the “Huatuo's Zhongzang Classic” in the eastern Han Dynasty, the “Chinese Pharmacopeia,” national processing norms, and local processing norm. PM was primary processed in the pre-Tang Dynasty, steamed or cooked with black bean juice in the Tang Dynasty, and repeatedly steamed and sun-dried nine times in the Song and Qing Dynasty. The processing of PM has evolved from simple to complex, which indicates that people's understanding of the nourishing effect of PM continues to improve. The attenuating effects and efficiency of PM processing are constantly improving and have received increasing attention [108, 109]. The continuous improvement of PM processing also indicates that there is a connection between the adverse reactions of PM and its processing. Modern research also proves that the toxicity of PM is significantly reduced after processing [29, 110–112]. Therefore, processing is an important factor that impacts the effect of PM.

However, the processing of PM has changed from complicated to simple in modern times; for example, PM processing was simplified from nine cycles of steaming and sun-drying to steaming once without sun-drying. Additionally, the methods of PM processing vary among regions. There are many kinds of processing methods, including ancient processing and modern processing, such as boiling, steaming, exposure, frying, and roasting, which were without accessories; black beans, wine, vinegar, rice swill, and so on; nine cycles of steaming and sun-drying; high pressure processing, microwave processing, fermentation processing, extrusion technology, etc. [109, 113]. Due to the diversity of processing norms, the types and contents of the chemical components in different processed products are different, which results in safety hazards in the clinical use of PM. The processing of PM could be combined with modern science and technology, such as the use of modern machinery and equipment (drum type frying machine, vacuum drying box) to achieve the homogeneity and stability of processing conditions. Furthermore, modern detection methods (HPLC, UV, GC, etc.) are used to achieve real-time quality monitoring.

In addition, researches have also speculated that the liver injury induced by PM might be related to an imbalance in intestinal flora [114], immune-specific effects, or idiosyncratic hepatotoxicity [115, 116].

#### 3.4.3. Metals and Elements Were Associated with Liver Injury

During the process of planting medicinal materials, planting conditions such as sunlight, moisture, climate, and soil should be fully guaranteed. However, with the development of agriculture, organophosphorus pesticides and heavy metal residues have become a major environmental factor affecting the quality of Chinese herbal medicine. The heavy metals that cause environmental and soil pollution mainly include mercury, cadmium, lead, chromium, and arsenic.

Recent studies have also demonstrated that heavy metal and element concentrations are associated with PM-induced liver injury [117, 118]. Reports have also shown that heavy metal contamination may lead to adverse reaction [119]. PM has many kinds of habitats, and different habitats have different environmental conditions such as temperature and humidity. Thus, the components and inorganic elements, and even heavy metal residues of PM, are correlated to geographical, climatic, and soil factors [120, 121]. Therefore, it is necessary to strengthen pharmacognosy traceability and quality control in order to prevent and reduce adverse reactions.

## 4. Discussion

**Figure 1:**
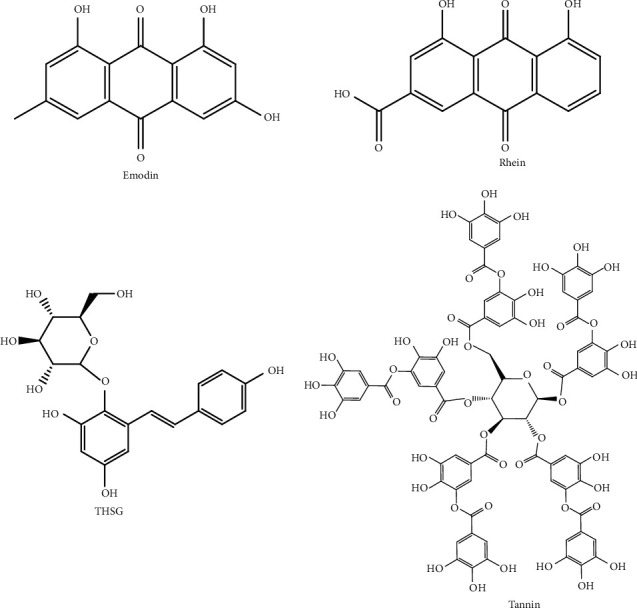
The structures of potential hepatotoxic compounds in PM.

According to the literature, the potential chemical composition and mechanism of liver injury caused by PM are summarized. The hepatotoxic compounds in PM (Figure 1) and the mechanism of the liver injury caused by PM need further study due to the integrity, complexity, and multitargeted and synergistic effects of this natural product.

First, as cholestasis in the body stimulates oxidative stress, the level of reactive oxygen species in the body increases, causing a series of reactions that lead to liver cell damage. Modern pharmacological studies have shown that many of the active ingredients in Chinese herbal medicines have antioxidation activity, and that they directly or indirectly reduce or eliminate the liver injury caused by cholestasis. For example, curcumin, a natural antioxidant in turmeric, plays an important role in the liver damage model caused by cholestasis by reducing the level of the inflammatory factor TNF-*α* in the liver [122, 123]. Additionally, the active ingredients in herbs associated with liver injury have been reported to directly or indirectly cause oxidative damage to the body, resulting in hepatotoxicity, such as *Melia toosendan*, *Dioscorea bulbifera* L., and *Radix Bupleuri* [124, 125], and causing liver damage by inducing oxidative stress in the body. What is the mechanism of the liver injury caused by PM? The potential hepatotoxic components of PM can cause oxidative stress by destroying mitochondrial function, leading to cholestasis and then resulting in liver injury, or the potential hepatotoxic components of PM can affect the synthesis, transport, secretion, etc. of bile acid, causing cholestasis, which in turn disturbs oxidative balance in the body, leading to liver injury. These mechanisms need to be further studied.

Second, the clinical manifestation of liver injury caused by PM is the accumulation of bile acid in the liver. The accumulation of bile acid in the liver is considered to be the leading cause of cholestasis-induced liver injury. Any failure in the synthesis, transport, secretion, etc. of bile acids may cause cholestasis. There are two ways to synthesize bile acids in liver cells: the classic pathway, catalyzed by cholesterol 7*α*-hydroxylase (CYP7A1), and the alternative pathway, catalyzed by sterol 27*α*-hydroxylase. However, the alternative route is usually activated when the classic pathway is blocked. CYP3A is a key CYP450 enzyme that breaks down bile acids [126]. After bile acid is synthesized, it is secreted by active transport; approximately 95% of bile acids rely on a variety of transporters and energy to enter the liver and intestinal circulation. A variety of protein molecules with transport functions present on hepatocytes, bile duct cell membranes, and small intestinal cell membranes, such as Ntcp, Bsep Mrp2, Asbt, etc., directly affect the transport and secretion of bile acids [127]. Certain chemical constituents of PM function by inhibiting the expression of these transporters or by mutating these transporters, thus causing cholestasis [128]. In addition, as described above, cholestasis in vivo can induce an inflammatory response and the production of inflammatory factors. Studies have shown that intracellular LPS and some inflammatory factors can rapidly reduce the expression of Ntcp on hepatocytes, of which IL-1 is the most effective [129, 130]. Notably, emodin and hydroxyemodin can inhibit the activity of Mrp2 according to reports. Is this the cause of liver damage in PM? Do the other medicinal substances in PM affect the activity of certain bile acid synthases or transport proteins; cause cholestasis in the synthesis, transport, and secretion of bile acids; and then promote liver injury?

Moreover, the degree of cholestasis-induced liver injury caused by PM processed with black bean was significantly decreased compared to that caused by PM without auxiliary materials (steamed PM). Additionally, steamed PM has a strong inhibitory effect on the activity of CYP2E1 [90, 131]. Black beans are common auxiliary materials in PM processing, and they are rich in protein, unsaturated fatty acids, vitamins, and phenolic acids, which have antioxidant and immunomodulatory effects [132, 133]. PM processed with black bean has been used since ancient times and is included in the Pharmacopeia. However, whether changes in the chemical composition and content of PM occur after processing with black beans is uncertain. The mechanism of the positive effect of PM processed with black beans is unknown. Moreover, can black beans enhance the antioxidant activity of PM when PM is processed with black beans? Do the ingredients in black beans participate in the synthesis, transport, and secretion of bile acids? Is the attenuation of liver injury caused by PM exerted through these pathways? Is the mechanism of the positive effects of PM processed with black bean related to these factors? These questions require further study.

For example, as described in Section 1, researchers have speculated that the hepatotoxic compound in PM may be the emodin. However, emodin has strong antibacterial and anti-inflammatory [124] activities [134] and inhibits the expression of inflammatory factors in the liver [135, 136]. Additionally, emodin also has a certain antioxidant effect, which can reduce the level of oxidative stress in the liver and play a role in liver protection [137]. Researchers found that prepared rhubarb has a significant therapeutic effect on liver injury in animals, but it has certain hepatotoxic effects on normal animals. Thus, researchers have proposed the symptom-based prescription theory [138]. Based on the theory, the hepatotoxicity of PM was studied, and a high dosage of PM had a toxic effect on normal rats and a therapeutic effect on rats with chronic liver injury [139]. Thus, if emodin is the hepatotoxic component in PM, perhaps it is possible to study the hepatotoxic component in PM based on the symptom-based prescription theory.

## 5. Conclusion

PM has been widely used because of its tonifying function. There are 380 Chinese patent medicines containing PM. With the increasing number of clinical cases of adverse drug reactions caused by PM and its preparations, its safety issues have aroused great attention of domestic and foreign researchers and drug regulatory authorities. Thus, the liver injury of PM has become a practical problem that seriously affects the safety of its clinical medication and needs to be solved urgently. However, so far, there are still controversies about the liver damage components and mechanisms of PM. Research will be necessary for further understanding of the hepatotoxicity induced by PM so as to take reasonable and effective measures to prevent it. In addition, the clinical features of liver injury that result from PM include cholestasis hepatitis. Besides, the AQs in PM could interfere with bile acid metabolism according to the references. PM should be avoided in combination with drugs that can cause cholestatic liver injury such as chlorpromazine and rifampicin.

## Figures and Tables

**Table 1:** Retrospective analysis on cases of liver injury of *Polygonum multiflorum*.

Case	Age	Duration of intake, day	Preparations	Presenting PM	Extrahepatic manifestation	Type of liver injury	Reference
264	5～63	7～365	Shou Wu tablet, Yangxue Shengfa capsule, Yanshou tablet, Jingwu capsule, Yishen Wufa oral liquid, Qibao Meiran pill, Tianma Shouwu tablet, Huo Li Su oral liquid, Geng Nian An tablet, Kunbao pill, Xinyuan capsule, Zhi Shou Wu granule, Renshen Shouwu capsule, Fu Fang Shou Wu oral liquid, Shou Wu pill, Anshen Bunao liquid, Jiang Zhi Ling tablet, Bantu pill, Bushen Yishou capsule, Huichun Ruyi capsule, Shenbao tablet, Jiangzhi Huazhuo capsule, Heishou Shengfa granule, Baishi pill, Shou Wu tea, etc.	PPM	Fever, fatigue, loss of appetite, jaundice, erythra, myalgia, nausea and vomiting, etc.	Mixed, hepatocellular, cholestasis	[10, 11]

214	19～78	3～120	He Shou Wu powder, He Shou Wu tea, He Shou Wu wine, etc.	Raw PM	Fever, fatigue, loss of appetite, jaundice, erythra, myalgia, nausea and vomiting, ascites, liver-palms, gray stool, etc.	Mixed, hepatocellular, cholestasis	[9, 12]

5	36～60	30～90	Runzao Zhiyang capsule	raw PM and PPM	Fever, fatigue, loss of appetite, jaundice, erythra, myalgia, nausea and vomiting, ascites, liver-palms, gray stool, etc.	Mixed, hepatocellular, cholestasis	[10, 13]

326	19～70	5～180	Huangjing Zanyu capsule, Huichun Ruyi capsule, Shenjiao capsule, He Shou Wu powder, He Shou Wu tea, He Shou Wu wine, decoction containing PM, herbal paste, healthcare product, etc.	PM	Fever, fatigue, loss of appetite, jaundice, erythra, myalgia, nausea and vomiting, ascites, liver-palms, gray stool, abdominal distension, etc.	Mixed, hepatocellular, cholestasis	[7, 14]

*Note*. PM means *Polygonum multiflorum*; the literature does not clearly indicate whether it is raw PM or PPM.

## Data Availability

The data used to support the findings of this study are available from the corresponding author upon request.
